# Supplementary material for: Nanoscale imaging of bacterial infections by sphingolipid expansion microscopy
Source: Nat Commun. 2020 Dec 2;11:6173. doi: 10.1038/s41467-020-19897-1 (PMC7710728; doi:10.1038/s41467-020-19897-1)
Supplement: Supplementary file 2 — Reporting Summary [file 41467_2020_19897_MOESM2_ESM.pdf]

nature research

Corresponding author(s): Markus Sauer

Last updated by author(s): Oct 27, 2020

Reporting Summary

Nature Research wishes to improve the reproducibility of the work that we publish. This form provides structure for consistency and transparency in reporting. For further information on Nature Research policies, see Authors & Referees and the Editorial Policy Checklist .

Statistics

For all statistical analyses, confirm that the following items are present in the figure legend, table legend, main text, or Methods section.

n/a	Confirmed	
☐	☒	The exact sample size (*n*) for each experimental group/condition, given as a discrete number and unit of measurement
☐	☒	A statement on whether measurements were taken from distinct samples or whether the same sample was measured repeatedly
☒	☐	The statistical test(s) used AND whether they are one- or two-sided<br>*Only common tests should be described solely by name; describe more complex techniques in the Methods section.*
☐	☒	A description of all covariates tested
☒	☐	A description of any assumptions or corrections, such as tests of normality and adjustment for multiple comparisons
☐	☒	A full description of the statistical parameters including central tendency (e.g. means) or other basic estimates (e.g. regression coefficient) AND variation (e.g. standard deviation) or associated estimates of uncertainty (e.g. confidence intervals)
☒	☐	For null hypothesis testing, the test statistic (e.g. *F*, *t*, *r*) with confidence intervals, effect sizes, degrees of freedom and *P* value noted<br>*Give P values as exact values whenever suitable.*
☒	☐	For Bayesian analysis, information on the choice of priors and Markov chain Monte Carlo settings
☒	☐	For hierarchical and complex designs, identification of the appropriate level for tests and full reporting of outcomes
☒	☐	Estimates of effect sizes (e.g. Cohen's *d*, Pearson's *r*), indicating how they were calculated

Our web collection on statistics for biologists contains articles on many of the points above.

Software and code

Policy information about availability of computer code

Data collection	Zeiss Zen 12.0.1.362 2012 SP3 (black), Zeiss ELYRA S.1 SR-SIM, Leica LAS AF Version 2.7.3.9723
Data analysis	Fiji 1.51n, Zeiss ELYRA S.1 SR-SIM platform, Imaris 8.4.1

For manuscripts utilizing custom algorithms or software that are central to the research but not yet described in published literature, software must be made available to editors/reviewers. We strongly encourage code deposition in a community repository (e.g. GitHub). See the Nature Research guidelines for submitting code & software for further information.

Data

Policy information about availability of data

All manuscripts must include a data availability statement. This statement should provide the following information, where applicable:

- Accession codes, unique identifiers, or web links for publicly available datasets
- A list of figures that have associated raw data
- A description of any restrictions on data availability

The datasets generated during and/or analysed during the current study are made available. All figures have asscociated raw data that are available from the corresponding authors upon reasonable request.

Field-specific reporting

Please select the one below that is the best fit for your research. If you are not sure, read the appropriate sections before making your selection.

☒ Life sciences ☐ Behavioural & social sciences ☐ Ecological, evolutionary & environmental sciences

Life sciences study design

All studies must disclose on these points even when the disclosure is negative.

Sample size	Sample sizes were determined based on the estimates from preliminary experiments and similar studies in the previous manuscripts (Chang, Chen et al. 2017 – Iterative expansion microscopy, or Zwettler et al. 2020 - Molecular resolution imaging by post-labeling expansion single-molecule localization microscopy) so that reasonable statistical analyses could be conducted.
Data exclusions	For determination of the distance between the outer (OM) and inner membrane (IM) of bacteria, we investigated three different infected cells and selected only those bacteria whose orientation allowed us to visualize spatially separated OM and IM (i.e. frontal views of bacteria).
Replication	The number of independent experiments performed for each experiment is given in the Figure legends.
Randomization	This is not relevant for our study because we could image only those cells that were localized close to the coverslip in the expanded gel. All other cells withdraw themselves from analysis.
Blinding	Blinding was not relevant to this study for the same reasons as specified above.

Reporting for specific materials, systems and methods

We require information from authors about some types of materials, experimental systems and methods used in many studies. Here, indicate whether each material, system or method listed is relevant to your study. If you are not sure if a list item applies to your research, read the appropriate section before selecting a response.

Materials & experimental systems

n/a	Involved in the study	
☐	☒	Antibodies
☐	☒	Eukaryotic cell lines
☒	☐	Palaeontology
☒	☐	Animals and other organisms
☒	☐	Human research participants
☒	☐	Clinical data

Methods

n/a	Involved in the study	
☒	☐	ChIP-seq
☒	☐	Flow cytometry
☒	☐	MRI-based neuroimaging

Antibodies

Antibodies used

anti-HSP60 (Santa Cruz, sc-57840), anti-Neisseria gonorrhoeae (N0600-02,US biological) and Prx3 (Origene, TA322470), anti-mouse Atto 647N (Rockland, 610-156-121S), anti-LPS (BioRAD, MCA2718), anti-rabbit Atto 647N (Sigma, 40839), anti-Cert (ab72536, abcam), anti-beta-Actin (A5441, Sigma), anti mouse Cy3 (111-165-146, Dianova)

Validation

- anti-HSP60 (Santa Cruz, sc-57840): Suitable for immunoflorescence, immunoprecipitation, Western blot; raised against recombinant serovar A HSP 60 of Chlamydia trachomatis origin. Recommended for detection of C-terminal region of HSP 60.
- anti-Neisseria gonorrhoeae (N0600-02, US biological): Rabbit Polyclonal antibody. This antibody has been shown to work in applications such as: ELISA, and Immunofluorescence. Immunogene: Whole Neisseria gonorrhoeae; ATCC 31426.
- Prx3 (Origene, TA322470): domestic rabbit polyclonal antibody reactivity: human; application: western blot, immunohistochemistry. Immunogene: Synthetic peptide corresponding to a region derived from 242-256 amino acids of Human peroxiredoxin 3.
- anti-mouse Atto 647N (Rockland, 610-156-121S): Polyclonal Anti-MOUSE IgG (H&L) (GOAT) Antibody ATTO 647N Conjugated. Application: ELISA, immunofluorescence, Western blot.
- anti-LPS (BioRAD, MCA2718): Monoclonal mouse anti-chlamydia LPS antibody. Application: ELISA, immunofluorescence, Western blot, clone: CF 6J12.
- anti-rabbit Atto 647N (Sigma, 40839): Polyclonal Atto 647 goat anti-rabbit IgG (whole molecule). Application: ELISA, immunofluorescence, Western blot.
- anti-Cert (ab72536, abcam): Rabbit polyclonal to CERT; Suiteable for immunofluorescence and Western blot. Reacts with: Human Predicted to work with: Rabbit, Horse, Chicken, Guinea pig, Cow, Dog, Turkey, Pig, Xenopus laevis, Chimpanzee, Zebrafish, Rhesus monkey, Gorilla, Orangutan, Xenopus tropicalis. Immunogen: Synthetic peptide corresponding to Human CERT.
- anti-beta-Actin (A5441, Sigma): Monoclonal Anti-β-Actin antibody produced in mouse has been used for immunofluorescence staining, immunoblotting and immunohistochemistry. Immunogen slightly modified β-cytoplasmic actin N-terminal peptide, Ac-Asp-Asp-Asp-Ile-Ala-Ala-Leu-Val-Ile-Asp-Asn-Gly-Ser-Gly-Lys, conjugated to KLH.
- anti mouse Cy3 (111-165-146, Dianova): Polyclonal goat anti-mouse antibody conjugated with Cy3; Suitable for ELISA, Flow Cytometry, Immunocytochemistry, Immunofluorescence, Immunohistochemistry (frozen sections), Immunohistochemistry (IHC), Immunohistochemistry (Paraffin-embedded Sections)

Eukaryotic cell lines

Policy information about cell lines

Cell line source(s)	Hela229 (ATCC CCL-2.1tm), Chang (purchased from ATCC)
Authentication	None of the cell lines used in this study were authenticated in our lab, as they were directly purchased from the internationally credible vendors.
Mycoplasma contamination	In our laboratory, the contamination of mycoplasma was regularly examined by PCR, and found no contamination was detected while we conducted experiments concerning this work.
Commonly misidentified lines (See ICLAC register)	No commonly misidentified cell lines were used.
