# Supplementary material for: Nanoscale imaging of bacterial infections by sphingolipid expansion microscopy
Source: Nat Commun. 2020 Dec 2;11:6173. doi: 10.1038/s41467-020-19897-1 (PMC7710728; doi:10.1038/s41467-020-19897-1)
Supplement: Supplementary file 3 — Description of Additional Supplementary Files [file 41467_2020_19897_MOESM3_ESM.pdf]

File title: Supplementary Movie 1.
Description: 10x ExM SIM z-stack of Hela229 cells infected with *Chlamydia trachomatis* for 24 h, fed with α-NH_2-ω-N_3-C_6-ceramide, fixed, permeabilized and stained with DBCO-Alexa Fluor 488. Chlamydia are clearly located at the inclusion membrane. Scale bar, 10 µm.
