# Supplementary material for: Nanoscale imaging of bacterial infections by sphingolipid expansion microscopy
Source: Nat Commun. 2020 Dec 2;11:6173. doi: 10.1038/s41467-020-19897-1 (PMC7710728; doi:10.1038/s41467-020-19897-1)
Supplement: Supplementary file 5 — Supplementary Information [file 41467_2020_19897_MOESM5_ESM.pdf]

Supplementary Information

Nanoscale imaging of bacterial infections by sphingolipid expansion microscopy

Ralph Götz[1,#], Tobias C. Kunz[2,#], Julian Fink[3], Jan Schlegel[1], Franziska Solger[2], Jürgen Seibel[3], Vera Kozjak-Pavlovic[3], Thomas Rudel[2,*] and Markus Sauer[1,*]

[1]Department of Biotechnology and Biophysics, Biocenter, University of Würzburg, Am Hubland, 97074 Würzburg, Germany
[2]Department of Microbiology, Biocenter, University of Würzburg, Am Hubland, 97074 Würzburg, Germany
[3]Institute for Organic Chemistry, University of Würzburg, Am Hubland, 97074 Würzburg, Germany

[#]These authors contributed equally: Ralph Götz, Tobias C Kunz
*Corresponding authors, e-mail: thomas.rudel@biozentrum.uni-wuerzburg.de (T.R.), m.sauer@uni-wuerzburg.de (M.S.)

General Experimental Information

Commercially available chemical reagents, purchased from *Sigma-Aldrich*, *Alfa Aesar*, *TCI* and *ACROS*, were used as received without further purification. All solvents were distilled before usage and dried when needed by using standard procedures. Moisture-sensitive reactions were performed under nitrogen atmosphere. Analytical thin-layer chromatography (TLC) was performed using silica gel pre-coated aluminium plates with a thickness of 0.2 mm. The compounds were visualized with a ninhydrin stain solution (600 mg ninhydrin, 6 mL glacial acetic acid, 200 mL *n*-butanol). Liquid column chromatography purification was performed with silica gel 60 (40–63 μm mesh, *Macherey-Nagel*).

Nuclear magnetic resonance (NMR) spectra were recorded on a *Bruker* Avance III HD 400/600 at 295 K. Chemical shifts (δ) are given in parts per million (ppm) with respect to the solvent residual proton signal ($\delta(CDCl_3)$ = 7.26 ppm) for ^{1}H or the resonance signal ($\delta(CDCl_3)$ = 77.16 ppm) for ^{13}C. Coupling constants (J) are reported in Hertz (Hz) and the multiplicity is abbreviated as s (singlet), d (doublet), t (triplet), m (multiplet), dd (doublet of doublets), br s (broad singlet) etc. ^{15}N signals were taken from (^{1}H,^{15}N)-HMBC projection and are referenced to CH_3NO_2. Signal assignment was performed with additional information of DEPT135, (^{1}H,^{1}H)-COSY, (^{1}H,^{13}C)-HSQC and (^{1}H,^{13}C)-HMBC. Atom numbers do not refer to the IUPAC nomenclature.

Attenuated total reflection (ATR) infrared (IR) spectra were recorded with a *JASCO* FT/IR-4600 instrument equipped with an ATR PRO ONE unit.

High resolution mass spectrometry (HRMS) was performed with a *Bruker* Daltonics micrOTOF and micrOTOF-Q III (electrospray ionization, ESI) instrument.

Abbreviations

a. u., arbitrary units; aq., aqueous; Boc, *tert*-butoxycarbonyl; DMF, *N*,*N*-dimethyl-formamide; EtOAc, ethyl acetate; HATU, 1-[bis(dimethylamino)-methylene]-1*H*-1,2,3-triazolo[4,5-*b*]pyridinium 3-oxide hexafluorophosphate ; M, molarity; MeCN, acetoni-trile; NEt_3, triethylamine; sphingosine, (2*S*,3*R*,*E*)-2-aminooctadec-4-ene-1,3-diol; rt, room temperature; TFA, trifluoroacetic acid; Tf_2O,trifluoromethanesulfonic anhydride.

Supplementary Materials

Synthetic Procedure and Characterization

***N*²-(*tert*-butoxycarbonyl)-*N*⁶-diazo-L-lysine (2)**

To a suspension of NaN_3 (380 mg, 5.85 mml, 1.44 eq.) in MeCN (5 mL) was added Tf_2O (820 µL, 4.87 mmol, 1.20 eq.) dropwise at 0 °C. The mixture was stirred for 2.5 h at 0 °C and the resulting solution was then transferred to a mixture of (*tert*-butoxycarbonyl)-L-lysine (1) (1.00 g, 4.06 mmol, 1.00 eq.), NEt_3 (1.13 mL, 8.12 mmol, 2.00 eq.) and $CuSO_4$ (6.48 mg, 40.6 µmol, 0.01 eq.) in MeCN (10 mL) at 0 °C. The ice bath was removed and the reaction mixture was stirred at RT for 18 h. After the addition of H_2O (50 mL) and 1 M aq. HCl (20 mL), the aqueous phase was extracted with EtOAc (4 x 60 mL). The combined organic phases were dried ($MgSO_4$) and concentrated under reduced pressure. The yellow residue was purified by column chromatography on silica gel (CH_2Cl_2/MeOH 20:1) to give 2 (936 mg, 3.44 mmol, 85 %) as a colourless oil, which partly crystallized[1].

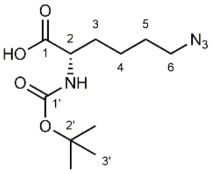

Formula: $C_{11}H_{20}N_4O_4$ (272.31 g/mol).

***R*f** (CH_2Cl_2/MeOH 30:1): 0.27.

¹H NMR ($CDCl_3$, 400 MHz): *δ* (2 rotamers) = 1.45 (s, 9H, *H*-3'), 1.46–1.57 (m, 2H, *H*-4), 1.57–1.66 (m, 2H, *H*-5), 1.66–1.80 (m, 1H, *H*-3), 1.80–1.96 (m, 1H, *H*-3), 3.29 (t, $^3J_{6,5}$ = 6.7 Hz, 2H, *H*-6), 4.07–4.40 (m, 1H, *H*-2), 5.06/6.55 (each br d, $^3J_{NH,2}$ = 8.1/5.5 Hz, together 1H, N*H*), 9.93 (br s, 1H, O*H*) ppm.

¹³C NMR ($CDCl_3$, 100 MHz): *δ* (2 rotamers) = 22.7 (*C*-4), 28.4 (3C, *C*-3'), 28.5 (*C*-5), 32.0/32.2 (*C*-3), 51.2 (*C*-6), 53.3/54.5 (*C*-2), 80.6/82.1 (*C*-2'), 155.8/157.0 (*C*-1'), 176.8/177.4 (*C*-1) ppm.

HRMS (ESI^+): *m/z* calcd. for $C_{11}H_{20}N_4NaO_4$ $[M+Na]^+$: 295.1377; found: 295.1383 ($|\Delta m/z|$ = 2.0 ppm); *m/z* calcd. for $C_{22}H_{40}N_8NaO_8$ $[2M+Na]^+$: 567.2861; found: 567.2866 ($|\Delta m/z|$ = 0.8 ppm).

FTIR (ATR): $\tilde{\nu}$ = 3313, 3186, 3073, 2979, 2936, 2870, 2095, 1712, 1686, 1514, 1479, 1456, 1392, 1368, 1281, 1234, 1189, 1150, 1106, 1051, 1024, 940, 852, 779, 753, 736, 643, 608, 564 cm^{-1}.

The measured spectroscopic data are in agreement with previously reported data[2-5].

***tert*-Butyl ((*S*)-6-azido-1-(((2*S*,3*R*,*E*)-1,3-dihydroxyoctadec-4-en-2-yl)amino)-1-oxohexan-2-yl)carbamate (3)**

To a solution of azido-acid **2** (45.5 mg, 167 µmol, 1.00 eq.) in dry DMF (3 mL) were added NEt_3 (69.9 µL, 501 µmol, 3.00 eq.) and HATU (69.8 mg, 184 µmol, 1.10 eq.) at 0 °C. After stirring at this temperature for 20 min, sphingosine (50.0 mg, 167 µmol, 1.00 eq.) and dry DMF (3 mL) were added. The ice bath was removed and the reaction mixture was stirred at rt for 3.5 h. After the addition of H_2O (10 mL) and saturated aq. NH_4Cl solution (30 mL), the aqueous layer was extracted with EtOAc (5 x 20 mL). The combined organic phases were washed with brine (20 mL), dried ($MgSO_4$) and concentrated under reduced pressure. The oily residue was purified by column chromatography on silica gel ($CHCl_3$/MeOH 40:1 to 30:1) to give **3** (44.1 mg, 79.6 µmol, 48 %) as a colourless, waxy solid.

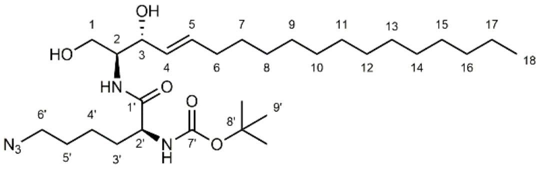

Formula: $C_{29}H_{55}N_5O_5$ (553.79 g/mol).

***R*f** (CH_2Cl_2/MeOH 30:1): 0.15.

^{1}H NMR ($CDCl_3$, 400 MHz): δ = 0.88 (t, $^3J_{18,17}$ = 6.9 Hz, 3H, *H*-18), 1.25–1.31 (m, 20H, *H*-8–17), 1.35–1.38 (m, 2H, *H*-7), 1.44 (s, 9H, *H*-9'), 1.48–1.90 (m, 6H, *H*-3'–5'), 2.03–2.08 (m, 2H, *H*-6), 3.29 (t, $^3J_{6',5'}$ = 6.7 Hz, 2H, *H*-6'), 3.71 (dd, $^2J_{1,1}$ = 11.6 Hz, $^3J_{1,2}$ = 3.3 Hz, 1H, *H*-1), 3.83–3.86 (m, 1H, *H*-2), 3.96 (dd, $^2J_{1,1}$ = 11.6 Hz, $^3J_{1,2}$ = 3.3 Hz, 1H, *H*-1), 3.99–4.04 (m, 1H, *H*-2'), 4.36–4.38 (m, 1H, *H*-3), 5.07 (d, $^3J_{NH,2'}$ = 6.9 Hz, 1H, N*H*), 5.51 (ddt, $^3J_{4,4}$ = 15.4 Hz, $^3J_{4,3}$ = 6.0 Hz, $^4J_{4,6}$ = 1.3 Hz, 1H, *H*-4), 5.80 (dtd, $^3J_{5,4}$ = 15.4 Hz, $^3J_{5,6}$ = 6.8 Hz, $^4J_{5,3}$ = 1.4 Hz, 1H, *H*-5), 6.86 (d, $^3J_{NH,2}$ = 8.0 Hz, 1H, N*H*) ppm.

^{13}C NMR ($CDCl_3$, 100 MHz): δ = 14.3 (*C*-18), 22.8 (*C*-17), 23.0 (*C*-4'), 28.4 (3C, *C*-9'), 28.6 (*C*-5'), 29.3 (*C*-7), 29.4, 29.5, 29.6, 29.8, 29.8, 29.8 (8C, *C*-8–15), 32.0 (2C, C-3' & C-16), 32.5 (*C*-6), 51.3 (*C*-6'), 54.6 (*C*-2), 55.1 (*C*-2'), 61.9 (*C*-1), 74.0 (*C*-3), 80.7 (*C*-8'), 128.6 (*C*-4), 134.2 (*C*-5), 156.2 (*C*-7'), 172.5 (*C*-1') ppm.

HRMS (ESI$^+$): *m/z* calcd. for $C_{29}H_{55}N_5NaO_5$ $[M+Na]^+$: 576.4095; found: 576.4073 ($|\Delta m/z|$ = 3.8 ppm); *m/z* calcd. for $C_{58}H_{110}N_{10}NaO_{10}$ $[2M+Na]^+$: 1129.8299; found: 1129.8274 ($|\Delta m/z|$ = 2.2 ppm).

(*S*)-2-Amino-6-azido-*N*-((2*S*,3*R*,*E*)-1,3-dihydroxyoctadec-4-en-2-yl)hexanamide / *α*-NH_2-*ω*-N_3-C_6-ceramide (4)

To a solution of carbamate **3** (37.0 mg, 6.68 µL) in CH_2Cl_2 (1 mL) was added TFA (200 µL) at 0 °C. The reaction mixture was stirred at this temperature for 2 h and was then quenched by the addition of H_2O (5 mL) and 1 M aq. NaOH (8 mL). After the extraction with EtOAc (5 x 10 mL), the combined organic phases were washed with brine (10 mL), dried ($MgSO_4$) and the solvent was removed under reduced pressure. The residue was purified by column chromatography on silica gel ($CHCl_3$/MeOH/25 % aq. NH_3 9:1:0.1) to give the crude product containing some NH_3 salts. To remove these impurities, the residue was dissolved in CH_2Cl_2 (15 mL) and washed with saturated aq. $NaHCO_3$ solution (10 mL). The aqueous phase was extracted with CH_2Cl_2 (6 x 5 mL), washed with brine (5 mL) and dried ($MgSO_4$). The solvent was removed under reduced pressure to give **4** (11.9 mg, 26.2 µmol, 39 %) as a colourless solid.

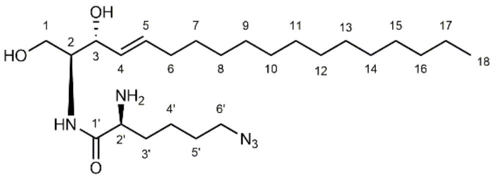

Formula: $C_{24}H_{47}N_5O_3$ (453.67 g/mol).

***R*f** ($CHCl_3$/MeOH/25 % aq. NH_3 9:1:0.1):0.26.

^{1}H NMR ($CDCl_3$, 600 MHz): δ = 0.87 (t, $^3J_{18,17}$ = 7.1 Hz), 1.25–1.30 (m, 20H, *H*-8–17), 1.33–1.37 (m, 2H, *H*-7), 1.43–1.52 (m, 2H, *H*-4'), 1.53–1.59 (m, 1H, *H*-3'), 1.59–1.67 (m, 2H, *H*-5'), 1.83–1.88 (m, 1H, *H*-3'), 2.03–2.07 (m, 2H, *H*-6), 2.21 (br s, 4H, 2 x O*H* & N*H*$_2$), 3.26–3.33 (m, 2H, *H*-6'), 3.39 (dd, $^3J_{2',3'}$ = 7.9 Hz, $^3J_{2',3'}$ = 4.7 Hz, 1H, *H*-2'), 3.71 (dd, $^2J_{1,1}$ = 11.4 Hz, $^3J_{1,2}$ = 3.5 Hz, 1H, *H*-1), 3.83–3.86 (m, 1H, *H*-2), 3.91 (dd, $^2J_{1,1}$ =11.4 Hz, $^3J_{1,2}$ =4.3 Hz, 1H, *H*-1), 4.30–4.32 (m, 1H, *H*-3), 7.82 (d, $^3J_{NH,2}$ = 7.7 Hz, 1H, N*H*), 5.52 (ddt, $^3J_{4,4}$ = 15.4 Hz, $^3J_{4,3}$ = 6.5 Hz, $^4J_{4,6}$ = 1.4 Hz, 1H, *H*-4), 5.78 (dtd, $^3J_{5,4}$ = 15.4 Hz, $^3J_{5,6}$ = 6.8 Hz, $^4J_{5,3}$ = 1.2 Hz, 1H, *H*-5) ppm.

^{13}C NMR ($CDCl_3$, 150 MHz): δ = 14.3 (*C*-18), 22.8 (*C*-17), 23.1 (*C*-4'), 28.8 (*C*-5'), 29.3 (*C*-7), 29.4, 29.5, 29.6, 29.8, 29.8, 29.8, 29.8 (8C, *C*-8–15), 32.1 (*C*-16), 32.5 (*C*-6), 34.8 (*C*-3'), 51.3 (*C*-6'), 55.0 (*C*-2), 55.3 (*C*-2'), 62.7 (*C*-1), 74.3 (*C*-3), 128.8 (*C*-4), 134.5 (*C*-5), 175.7 (*C*-1') ppm.

^{15}N NMR ($CDCl_3$, 60 MHz): δ = −352.3 (*N*H$_2$), −309.4 (C-*N*-N-N), −265.8 (*N*H), −132.8 (C-N-*N*-N) ppm.

HRMS (ESI$^+$): *m/z* calcd. for $C_{24}H_{47}N_5NaO_3$ [M+Na]$^+$: 476.35711; found: 476.35763 ($|\Delta m/z|$ = 1.08 ppm).

FTIR (ATR): $\tilde{\nu}$ = 3414, 3315, 3271, 3183, 3087, 2677, 2096, 1651, 1614, 1552, 1467, 1455, 1438, 1365, 1348, 1304, 1249, 1165, 1126, 1082, 1044, 1012, 992, 962, 932, 822, 801, 720, 801, 720, 650, 616, 590, 557 cm^{-1}.

NMR Spectra

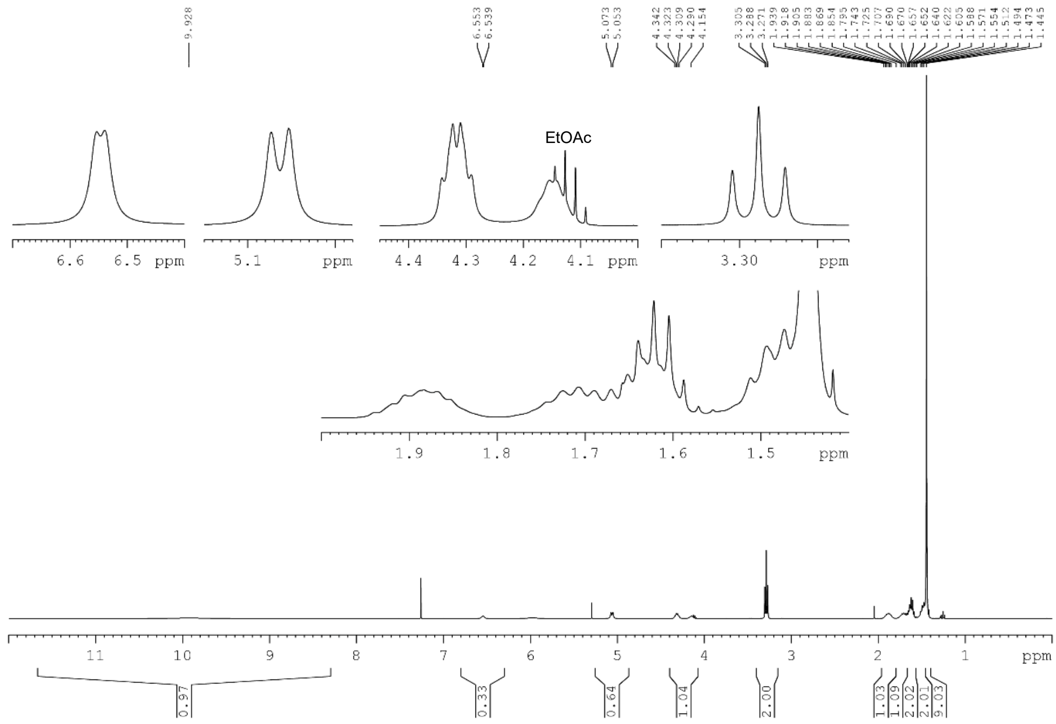

Supplementary Figure 1. ^{1}H NMR spectrum of **2** ($CDCl_3$, 400 MHz).

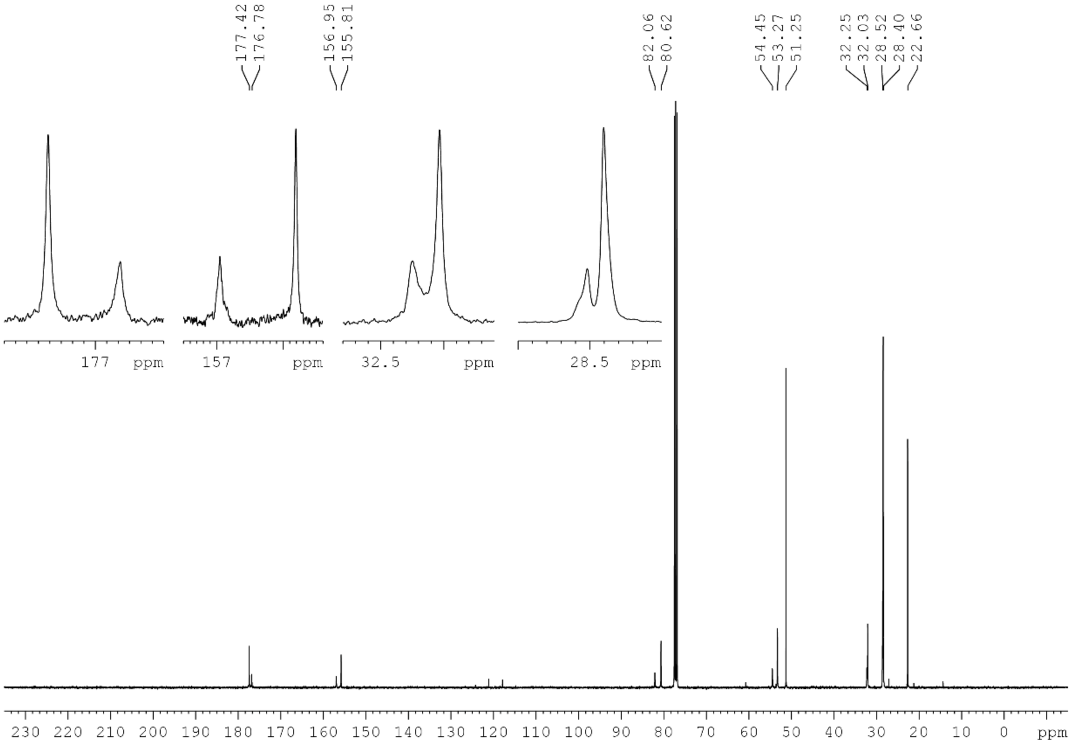

Supplementary Figure 2. ^{13}C NMR spectrum of **2** ($CDCl_3$, 100 MHz).

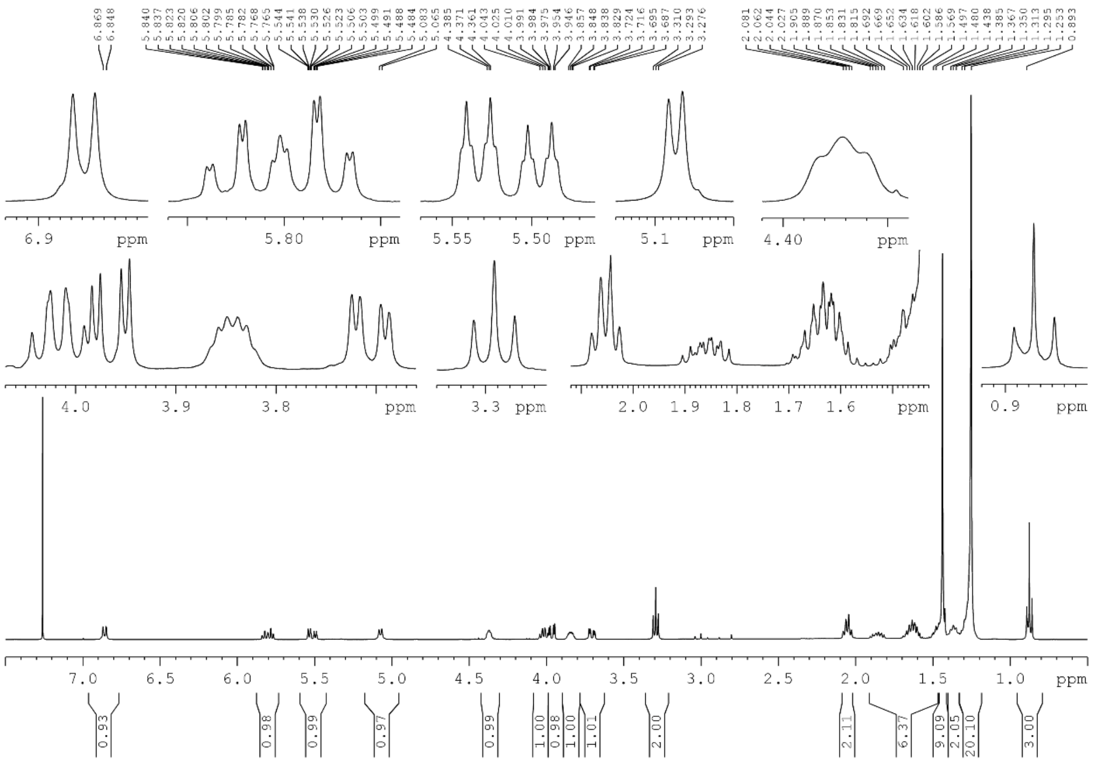

Supplementary Figure 3. ^{1}H NMR spectrum of **3** ($CDCl_3$, 400 MHz).

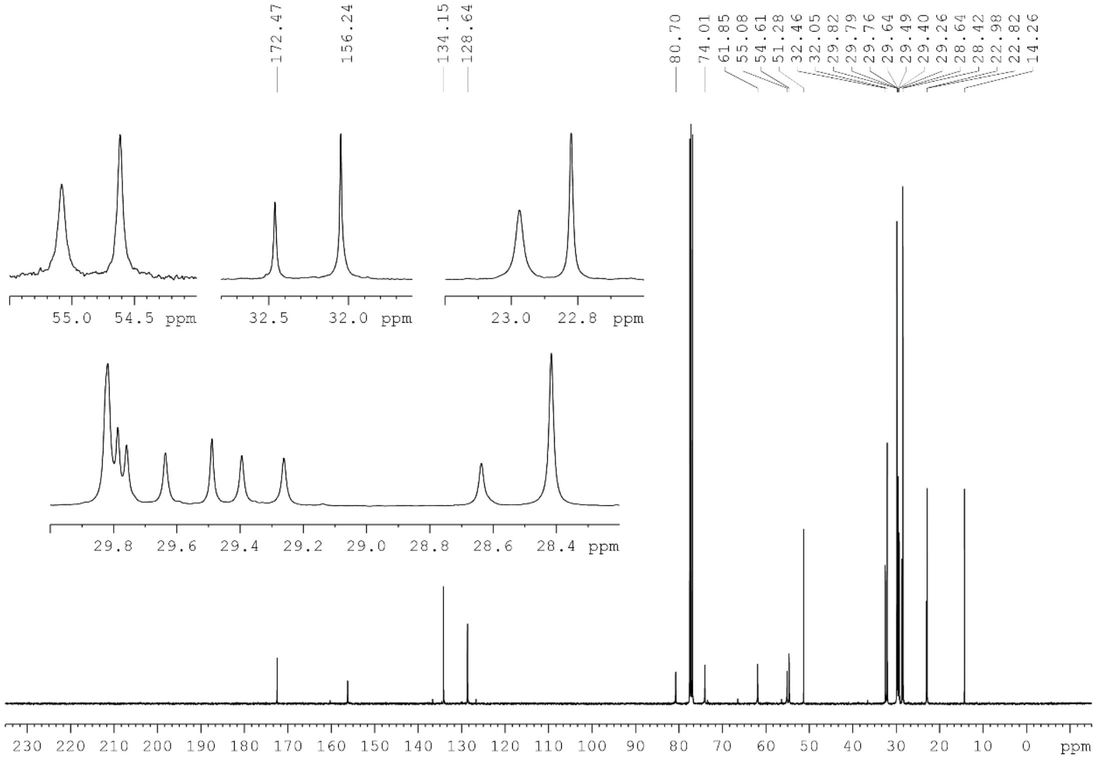

Supplementary Figure 4. ^{13}C NMR spectrum of **3** ($CDCl_3$, 100 MHz).

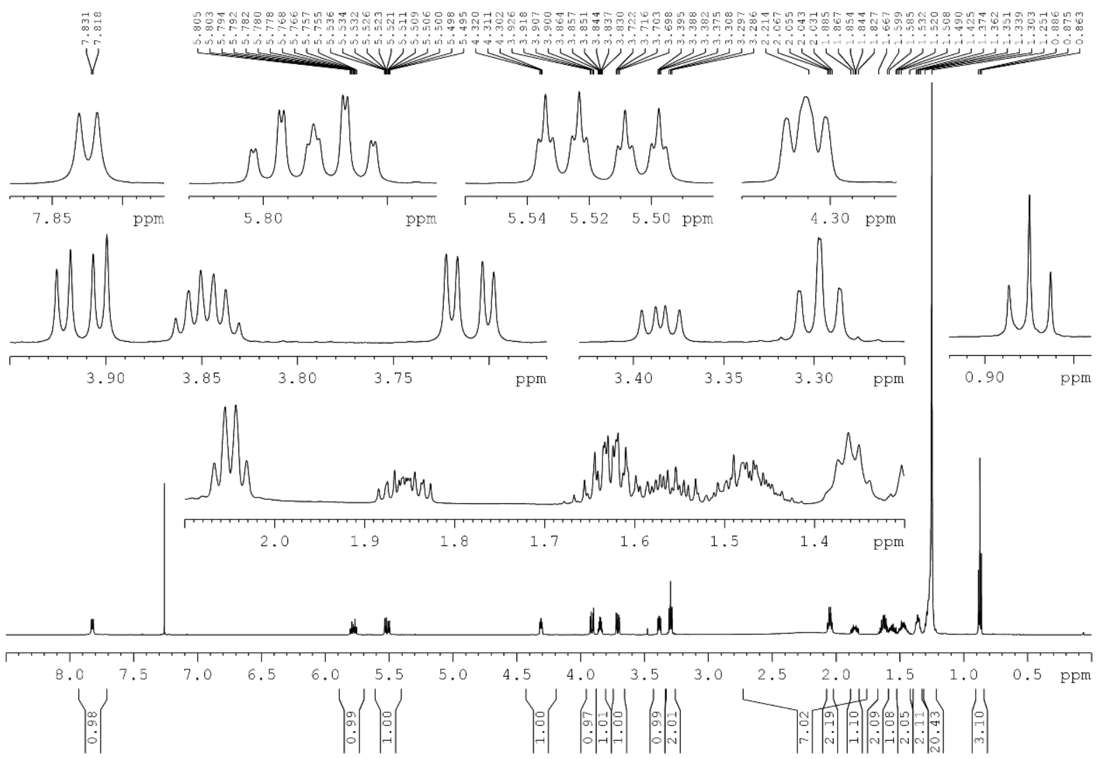

Supplementary Figure 5. ^1H NMR spectrum of **4** ($CDCl_3$, 600 MHz).

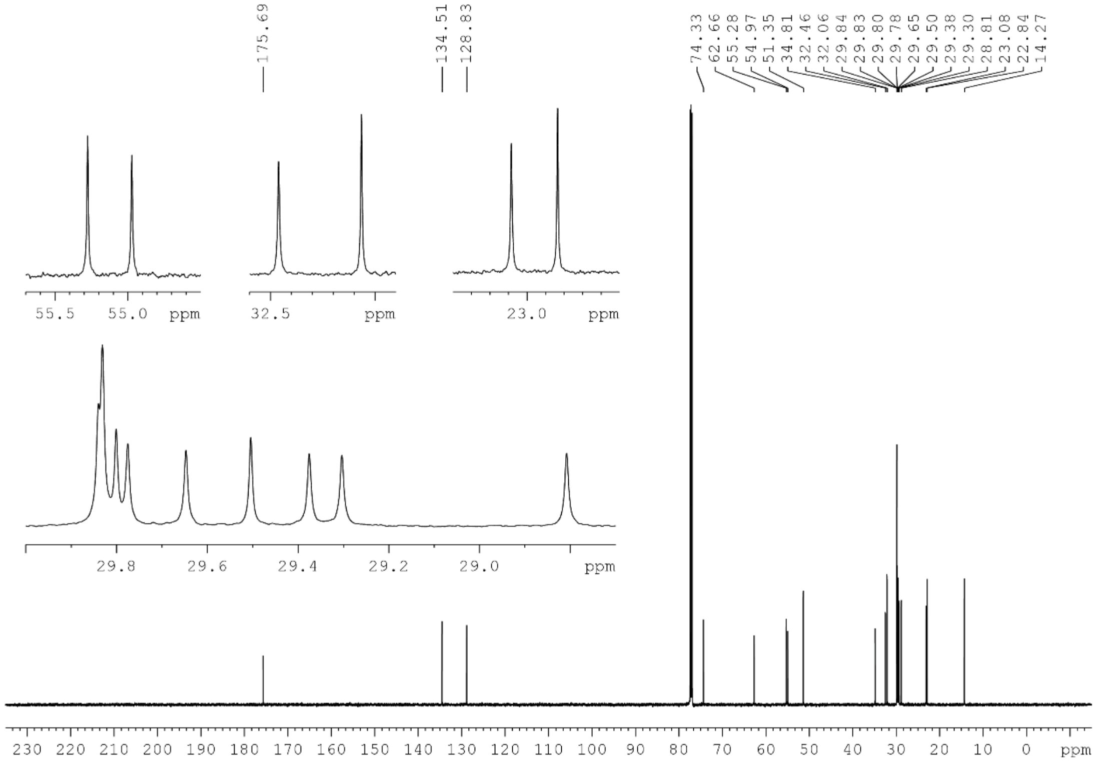

Supplementary Figure 6. ^{13}C NMR spectrum of **4** ($CDCl_3$, 150 MHz).

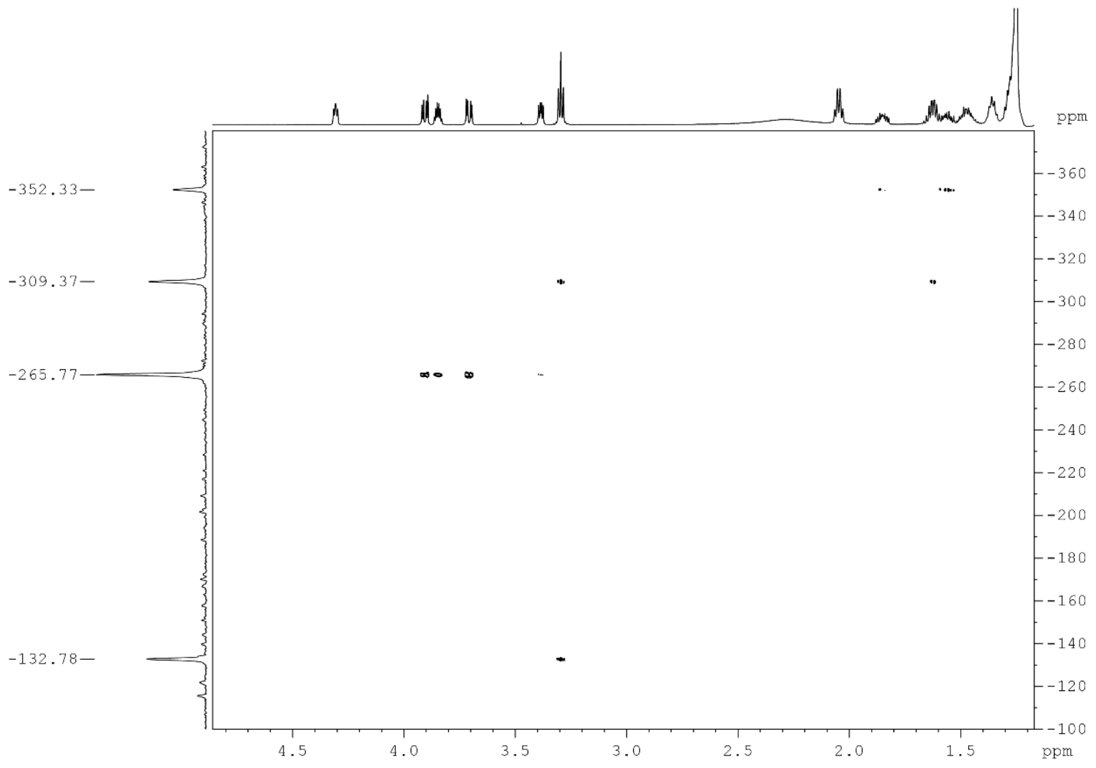

Supplementary Figure 7. (^1H,^{15}N)-HMBC NMR spectrum of **4** ($CDCl_3$, 600 MHz).

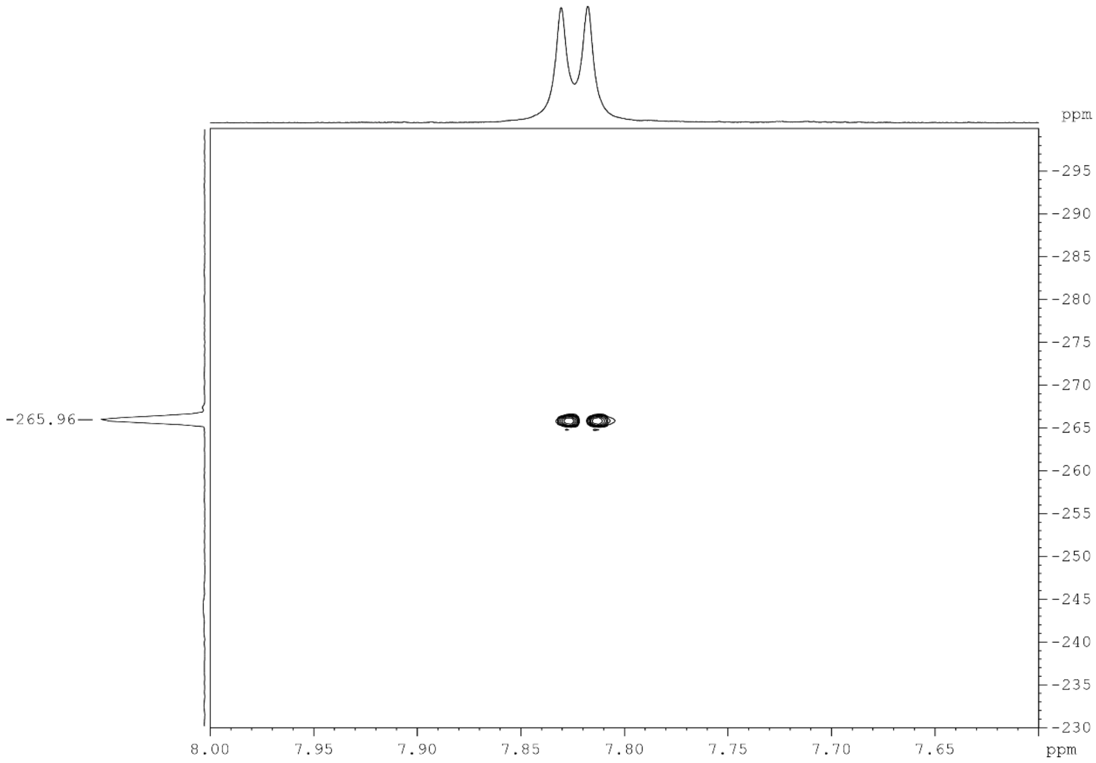

Supplementary Figure 8. (^{1}H,^{15}N)-HSQC NMR spectrum of **4** ($CDCl_3$, 600 MHz).

Mass Spectra

Compound Spectrum SmartFormula Report

Analysis Info

Analysis Name	D:\Data\spektren2019\24062019_SB080_SEI_44_01_4188.d	Acquisition Date	6/24/2019 3:42:37 PM
Method	Automation_esi_tune_pos_low.m	Operator	admin
Sample Name	24062019_SB080_SEI	Instrument	micrOTOF 213750.00088
Comment	in MeCN/CHCl3		

Acquisition Parameter

Source Type	ESI	Ion Polarity	Positive	Set Nebulizer	0.7 Bar
Focus	Not active	Set Capillary	4500 V	Set Dry Heater	200 °C
Scan Begin	50 m/z	Set End Plate Offset	-500 V	Set Dry Gas	5.0 l/min
Scan End	2000 m/z	n/a	n/a	Set Divert Valve	Source

+MS, 0.2-2.5min #8-100

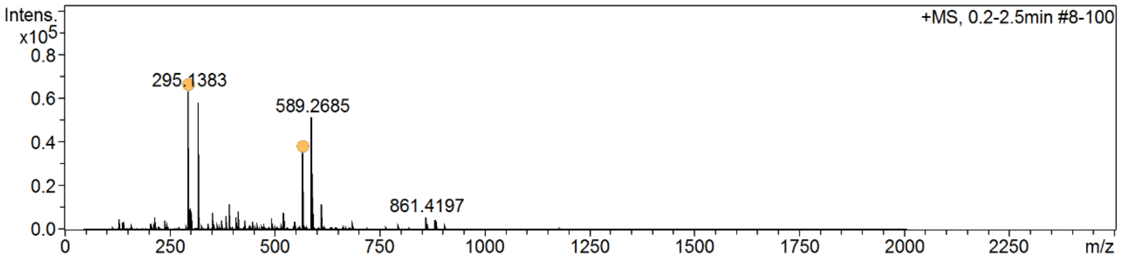

Meas. m/z	#	Ion Formula	m/z	err [ppm]	mSigma	# mSigma	Score	rdb	e⁻ Conf	N-Rule
295.1383	1	C11H20N4NaO4	295.1377	-2.0	4.1	1	100.00	3.5	even	ok
567.2866	1	C22H40N8NaO8	567.2861	-0.8	8.6	1	100.00	6.5	even	ok

Supplementary Figure 9. Mass spectrum of **2** (ESI$^+$).

Compound Spectrum SmartFormula Report

Analysis Info

Analysis Name	D:\Data\spektren2019\24062019_JF238_SEI_44_01_4177.d	Acquisition Date	6/24/2019 1:54:26 PM
Method	Automation_esi_tune_pos_mid.m	Operator	admin
Sample Name	24062019_JF238_SEI	Instrument	micrOTOF 213750.00088
Comment	in MeCN/CHCl3		

Acquisition Parameter

Source Type	ESI	Ion Polarity	Positive	Set Nebulizer	0.7 Bar
Focus	Not active	Set Capillary	4500 V	Set Dry Heater	200 °C
Scan Begin	150 m/z	Set End Plate Offset	-500 V	Set Dry Gas	5.0 l/min
Scan End	3500 m/z	n/a	n/a	Set Divert Valve	Source

+MS, 0.2-2.5min #12-154

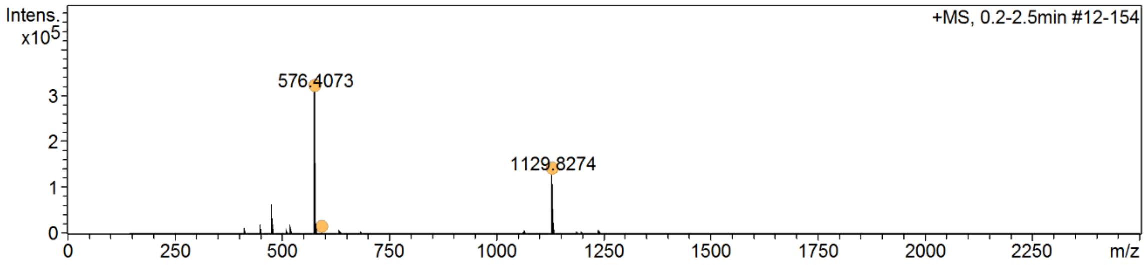

Meas. m/z	#	Ion Formula	m/z	err [ppm]	mSigma	# mSigma	Score	rdb	e⁻ Conf	N-Rule
576.4073	1	C29H55N5NaO5	576.4095	3.8	3.4	1	100.00	4.5	even	ok
592.3864	1	C29H55KN5O5	592.3835	-4.9	85.2	1	100.00	4.5	even	ok
1129.8274	1	C58H110N10NaO10	1129.8299	2.2	3.4	1	100.00	8.5	even	ok

Supplementary Figure 10. Mass spectrum of **3** (ESI$^+$).

Mass Spectrum SmartFormula Report

Analysis Info

Analysis Name	D:\Data\Spektren2019\2019_1505_SEI_JF241_3.d	Acquisition Date	7/5/2019 1:27:25 PM
Method	tune_pos_wide.m	Operator	Sebastian
Sample Name	2019_1505_SEI_JF241	Instrument	micrOTOF-Q III 8228888.20516
Comment	Fink Julian<br>JF241<br>5 pmol/ul in MeOH/CHCl3		

Acquisition Parameter

Source Type	ESI	Ion Polarity	Positive	Set Nebulizer	0.4 Bar
Focus	Not active	Set Funnel 1 RF	200.0 Vpp	Set Dry Heater	200 °C
Scan Begin	50 m/z	Set Funnel 2 RF	300.0 Vpp	Set Dry Gas	4.0 l/min
Scan End	4000 m/z	Set Hexapole RF	400.0 Vpp	Set Divert Valve	Waste

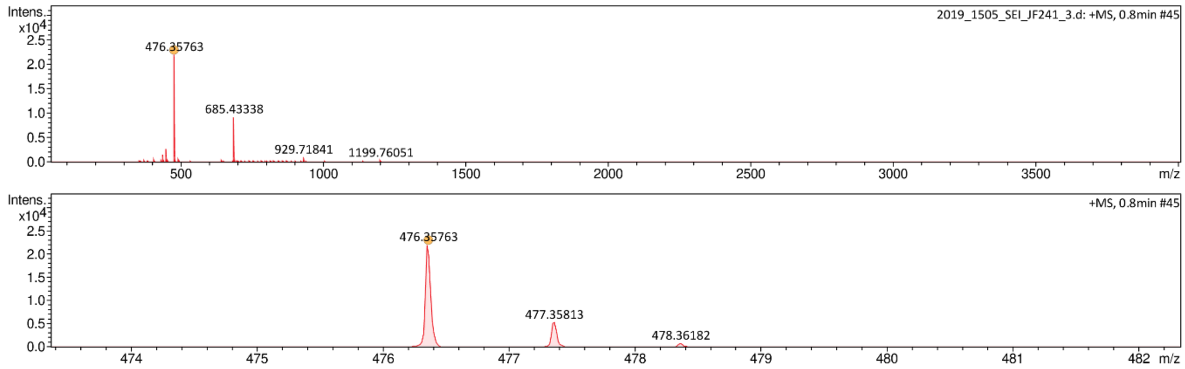

Meas. m/z	#	Ion Formula	m/z	err [ppm]	mSigma	# mSigma	Score	rdb	e⁻ Conf	N-Rule
476.35763	1	C24H47N5NaO3	476.35711	-1.08	26.7	1	100.00	3.5	even	ok

Bruker Compass DataAnalysis 4.2 printed: 7/5/2019 1:30:56 PM by: Sebastian Page 1 of 1

Supplementary Figure 11. Mass spectrum of **4** (ESI$^+$).

IR Spectra

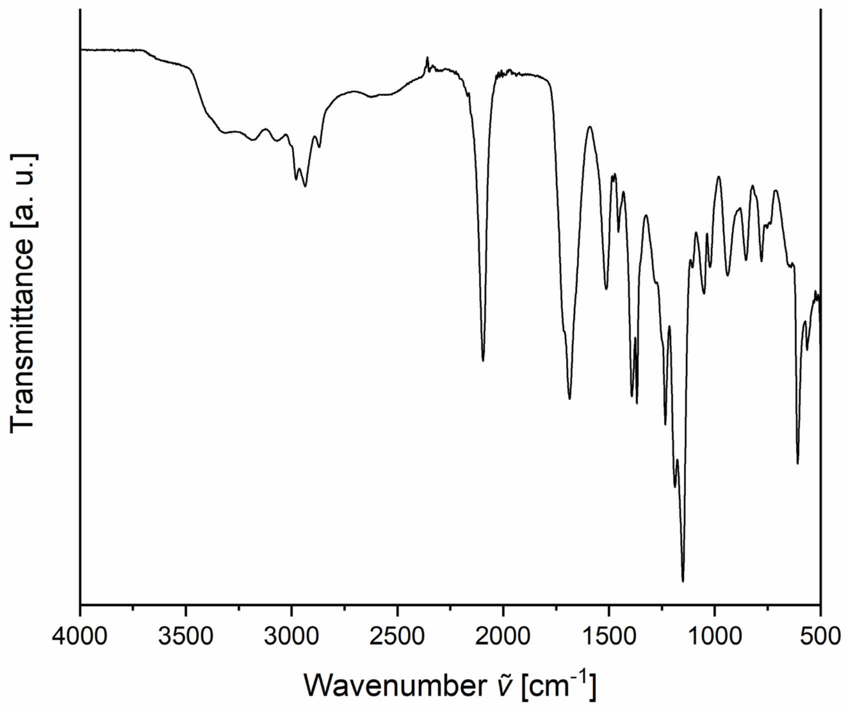

Supplementary Figure 12. FTIR spectrum of **2** (ATR).

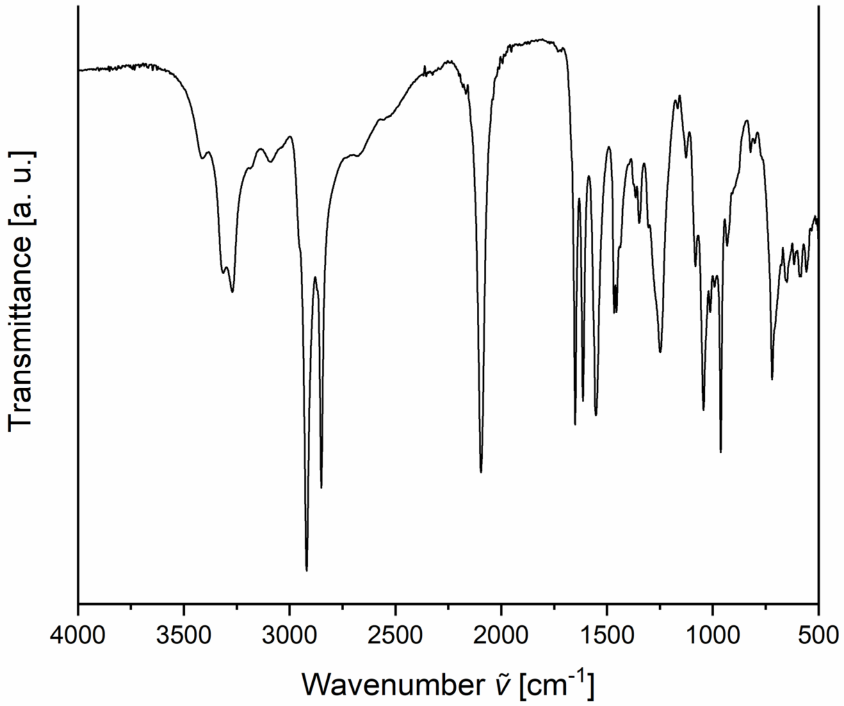

Supplementary Figure 13. FTIR spectrum of **4** (ATR).

ExM Images and Reference Experiments

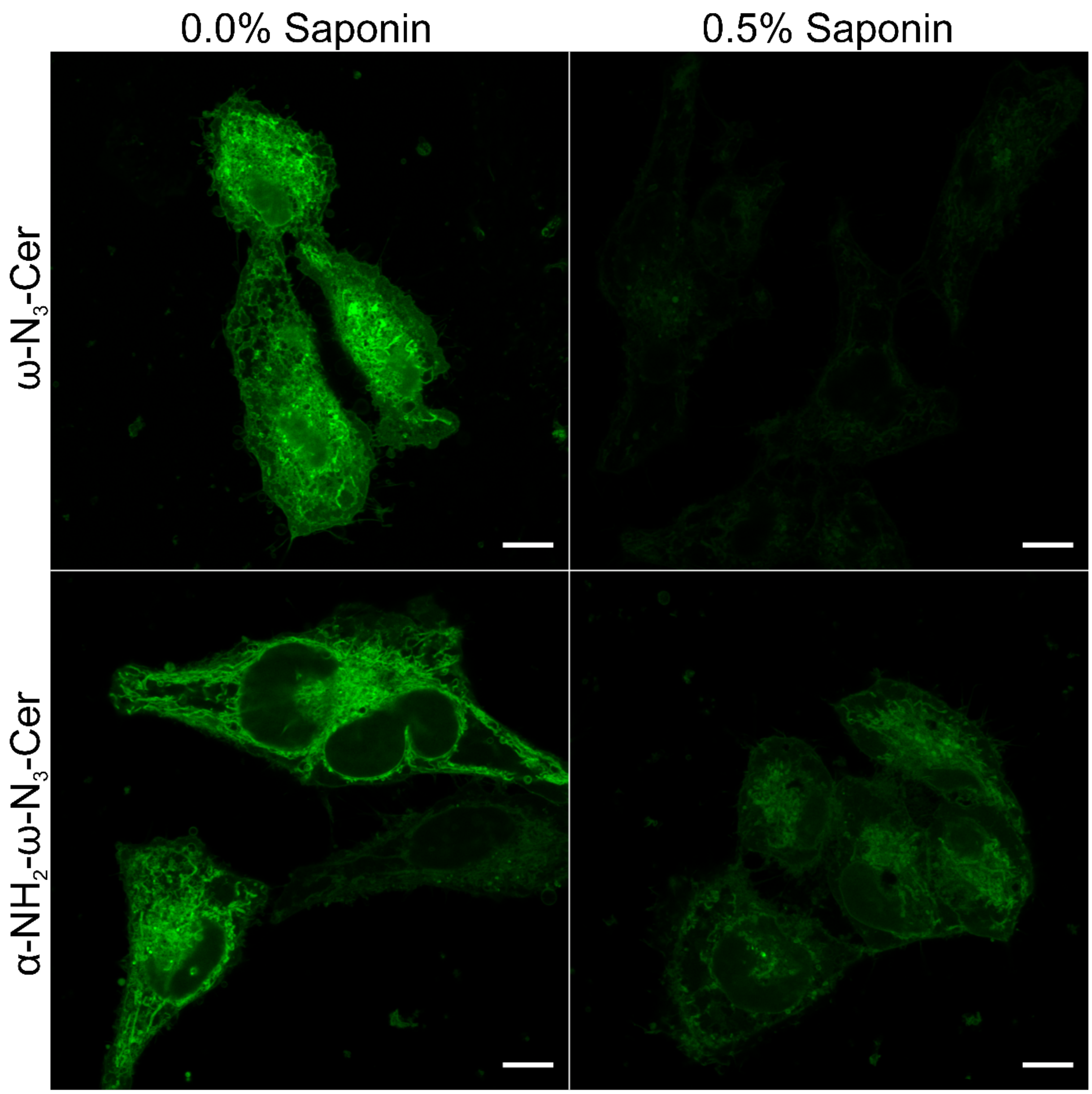

Supplementary Figure 14. Ceramide signal after treatment with saponin. Hela229 cells were fed with ω-N_3-C_6-ceramide or α-NH_2-ω-N_3-C_6-ceramide fixed, permeabilized with saponine and stained with DBCO-Alexa Fluor 488. Confocal fluorescence images show that ω-N_3-C_6-ceramide is efficiently washed out by addition 0.5% saponin while the NH_2-N_3-Cer signal remains preserved. The data were obtained from *n*=2 independent experiments. Scale bars, 10 µm.

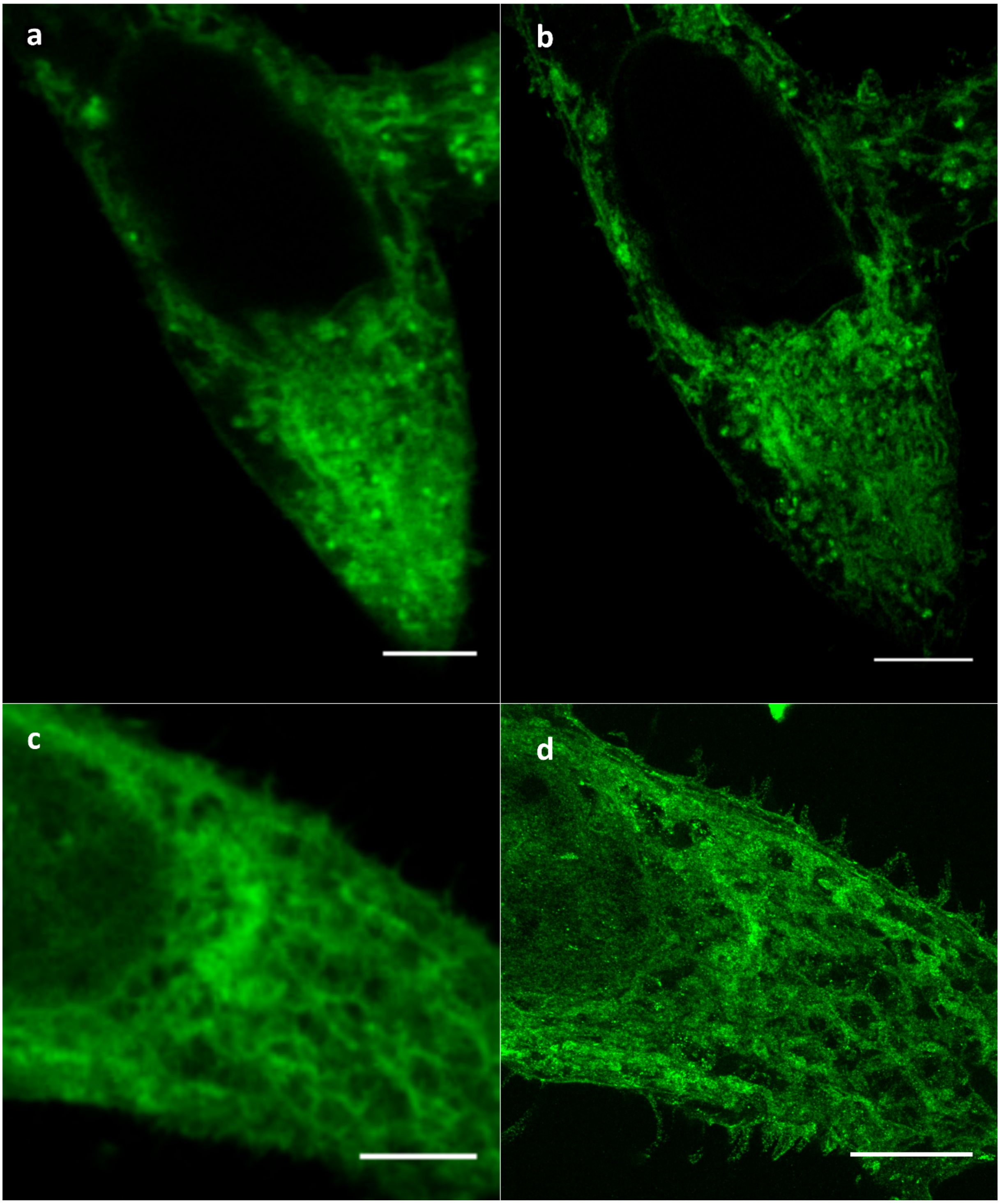

Supplementary Figure 15. Confocal fluorescence images of the same HeLa229 cells pre- (a,c) and post- 4x (b) and 10x (d) expansion. Cells were fed with α-NH_2-ω-N_3-C_6-ceramide (NH_2-N_3-Cer), fixed, permeabilized, stained with DBCO-Alexa Fluor 488 and gelated. The images demonstrate isotropic expansion. The effective expansion factors were determined to 4.1x and 9.8x, respectively, from the cell's diameters before and after expansion. The data were obtained from *n*=1 experiment. Scale bars, unexpanded 5 µm (a,c), 4x expanded 20 µm (b) and 10x expanded 50 µm (d).

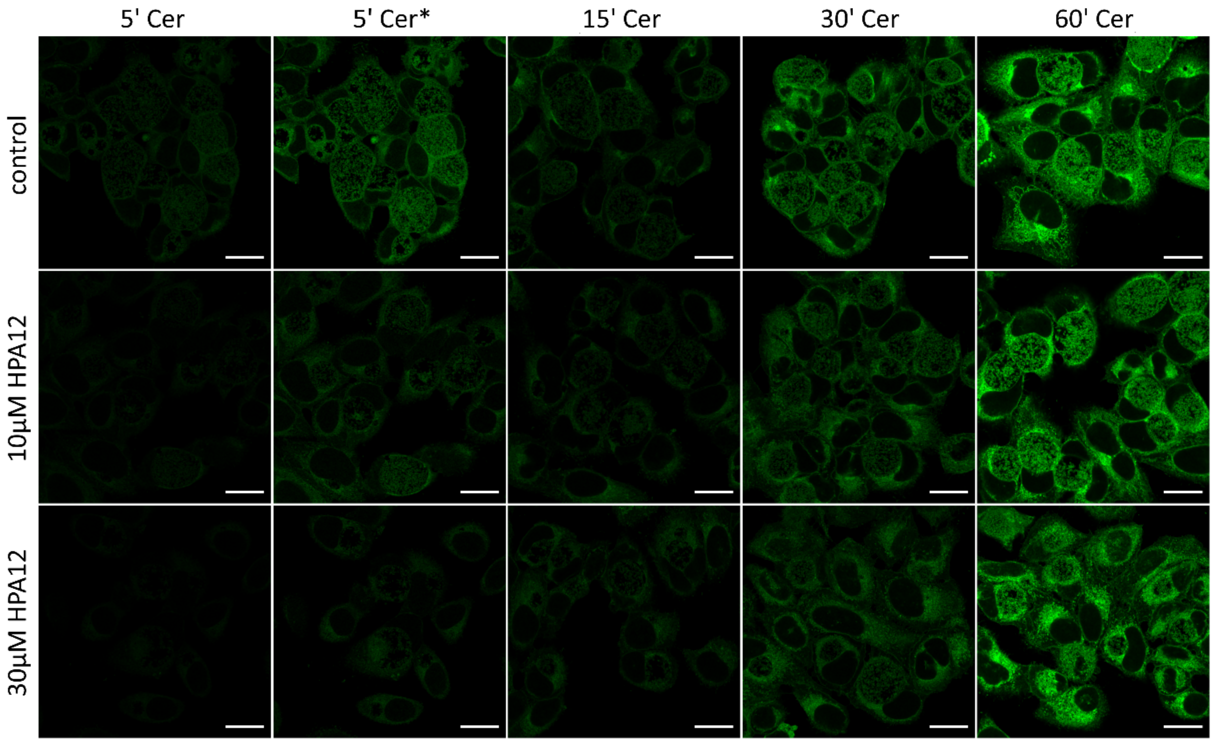

Supplementary Figure 16. HPA-12 inhibits the uptake of α-NH_2-ω-N_3-C_6-ceramide. HeLa229 cells were infected with *Chlamydia trachomatis* and treated with HPA-12 at 24 h of infection. 8 h later cells were fed with 10 µM α-NH_2-ω-N_3-C_6-ceramide for 5-60 min, fixed, permeabilized and stained with DBCO-Alexa Fluor 488 (green). Confocal fluorescence images show that uptake of ceramides is reduced during the first 5 -15 min, while little to no difference is observed at longer incubation times. The contrast of the images taken after 5 min (5' Cer*) was increased to highlight the differences after 5 min. The data were obtained from *n*=3 independent experiments. Scale bars, 20 µm.

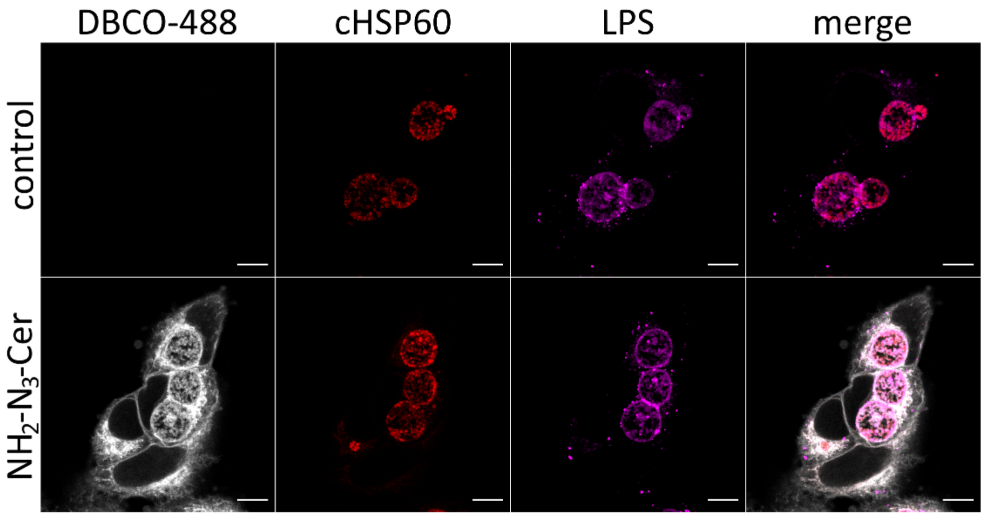

Supplementary Figure 17. α-NH2-ω-N3-C6-ceramide uptake does not influence chlamydial LPS levels. HeLa229 cells were infected with *Chlamydia trachomatis* and treated with 10 µM α-NH2-ω-N3-C6-ceramide for 60 min, fixed, permeabilized and stained with DBCO-Alexa Fluor 488 (gray), chlamydial HSP60 (red) and chlamydial LPS (magenta). Confocal fluorescence images show that uptake of α-NH2-ω-N3-C6-ceramide does not result in changes of LPS. The data were obtained from *n*=2 independent experiments. Scale bars, 10 µm.

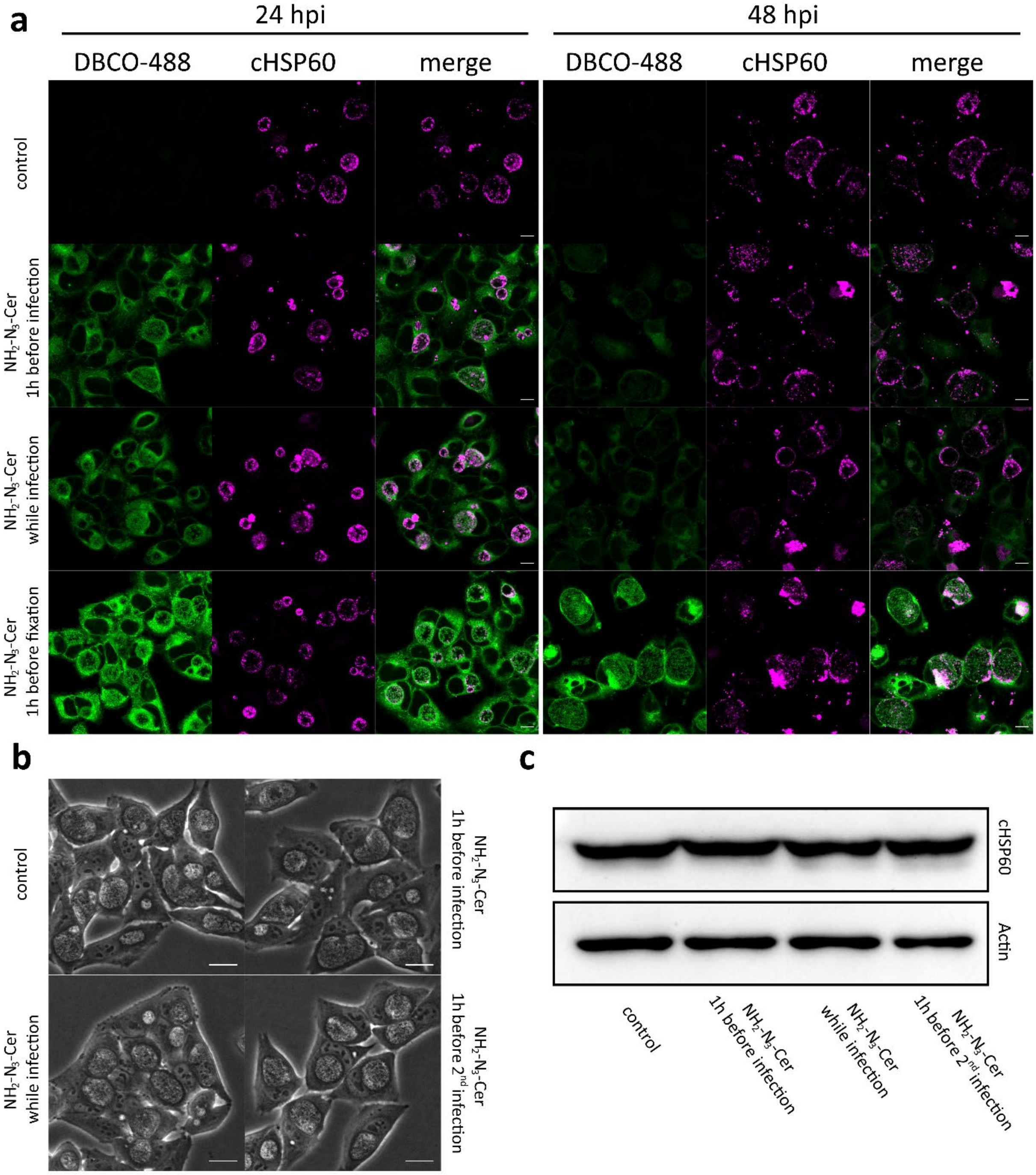

Supplementary Figure 18. α-NH_2-ω-N_3-C_6-ceramide is not influencing chlamydial development and progeny. (a) Hela229 cells infected with *Chlamydia trachomatis* for 24h and 48h were fed before infection, continuously while infection and before fixation with α-NH_2-ω-N_3-C_6-ceramide. The infected and fed cells were fixed, permeabilized and stained with DBCO-Alexa 488 (green) and the *Chlamydia trachomatis* antibody cHSP60 directed against chlamydial heat shock protein (magenta). (b,c) Secondary infection in Hela229 cells with the chlamydial progeny of Hela229 cells infected for 48 hours, fed before primary infection, continuously while primary infection and before secondary infection. Analysis by light microscopy (b) and Western Blot analysis (c). The data were obtained from *n*=2 independent experiments. Source data are provided as Source Data file. Scale bars, 10 µm (a), 20 µm (b).

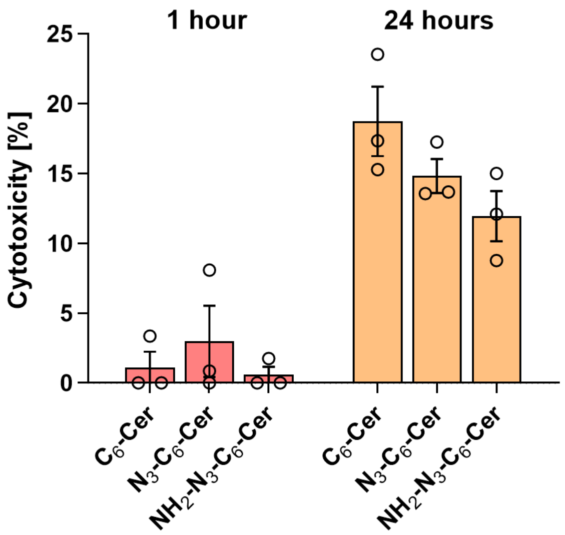

Supplementary Figure 19. LDH-assay of HeLa229 cells fed with C_6-ceramide, ω-N_3-C_6-ceramide or α-NH_2-ω-N_3-C_6-ceramide for 1 or 24 hours. Each column bar consists of n=3 biologically independent samples. Data are presented as mean values +/- SEM. The measurement of each biologically independent sample is represented as a circle. Source data are provided as Source Data file.

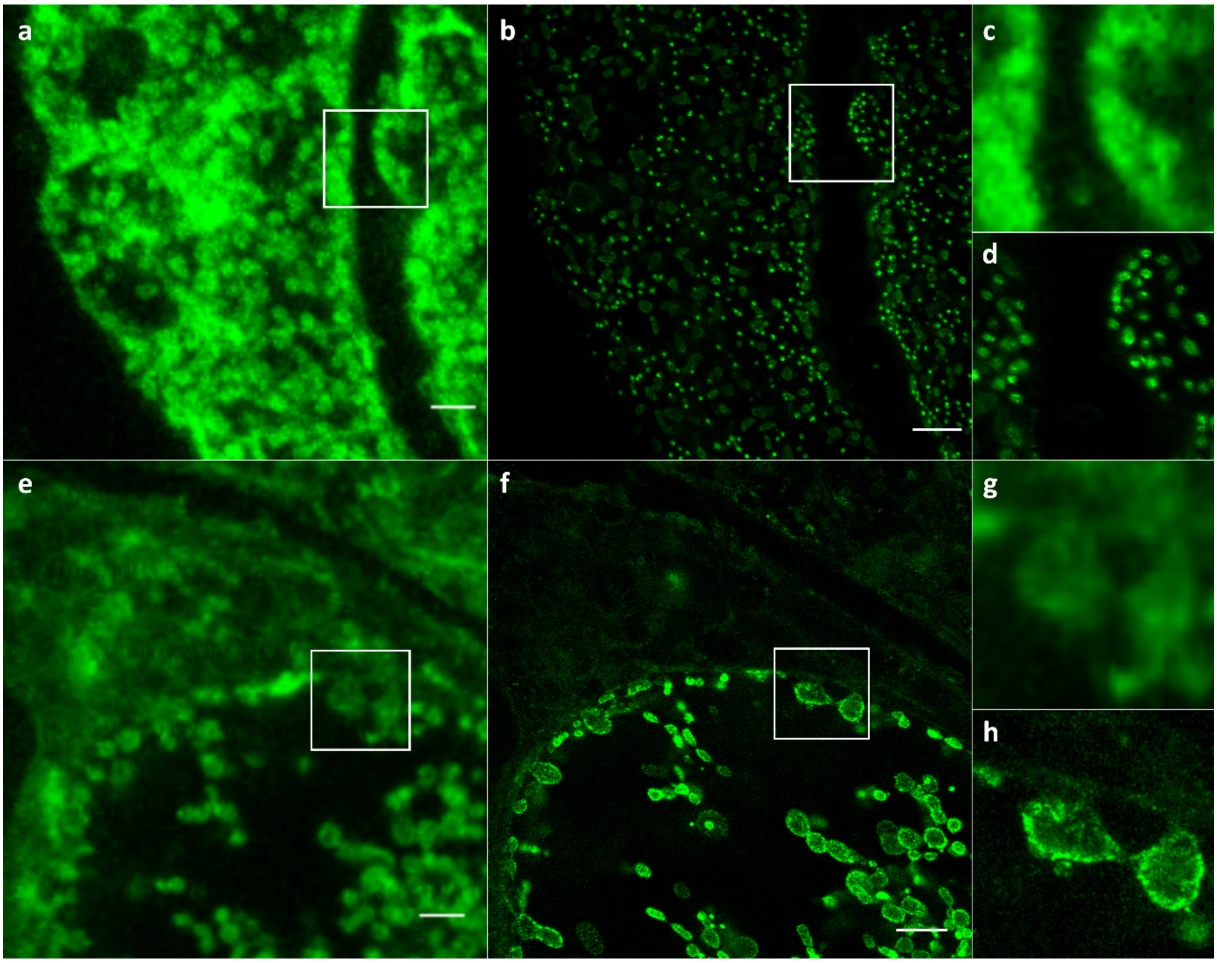

Supplementary Figure 20. Comparing pre- and post-expanded infected cells. Confocal fluorescence images of the same areas of HeLa229 cells infected with *S. negevensis* (a-d) or *C. trachomatis* (e-h), before (a,c,e,g) and after (b,d,f,h) 10x expansion. The infected cells were fed α-NH_2-ω-N_3-C_6-ceramide, fixed, permeabilized, and stained with DBCO-Alexa 488 following gelation. (c,d) and (g,h) show magnified views of the regions outlined by the white boxes in the main images. The data were obtained from n=1 experiment. Scale bars, unexpanded 2 µm, 10x expanded 20 µm.

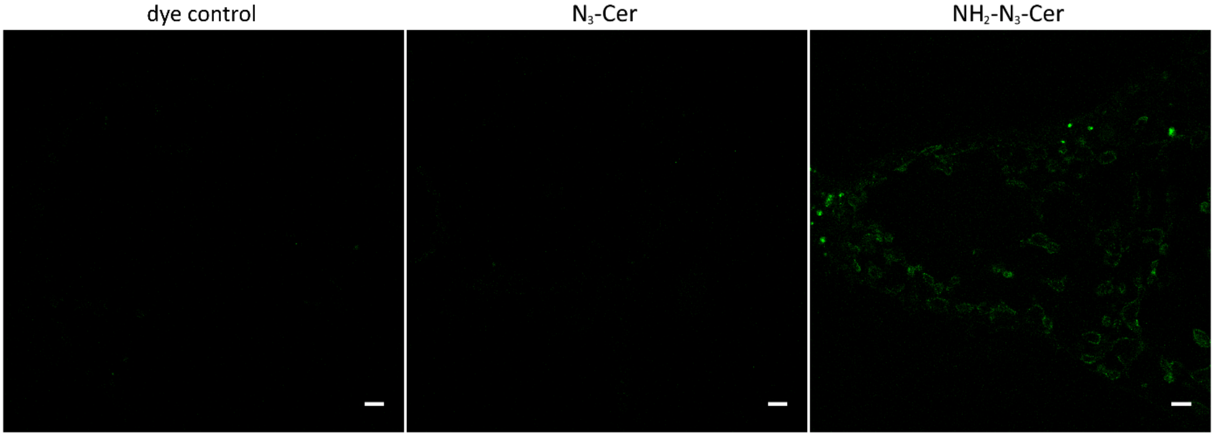

Supplementary Figure 21. Confocal fluorescence images of HeLa229 cells infected with *Chlamydia trachmomatis* for 24 h, without ceramide feeding (dye control), with ω-N_3-C_6-ceramide (N_3-Cer), or with α-NH_2-ω-N_3-C_6-ceramide (NH_2-N_3-Cer), fixed, permeabilized and stained with DBCO-Alexa Fluor 488. Only after feeding with α-NH_2-ω-N_3-C_6-ceramide (NH_2-N_3-Cer) the bacteria are visualized. The data were obtained from *n*=2 independent experiments. Scale bars, 10 µm.

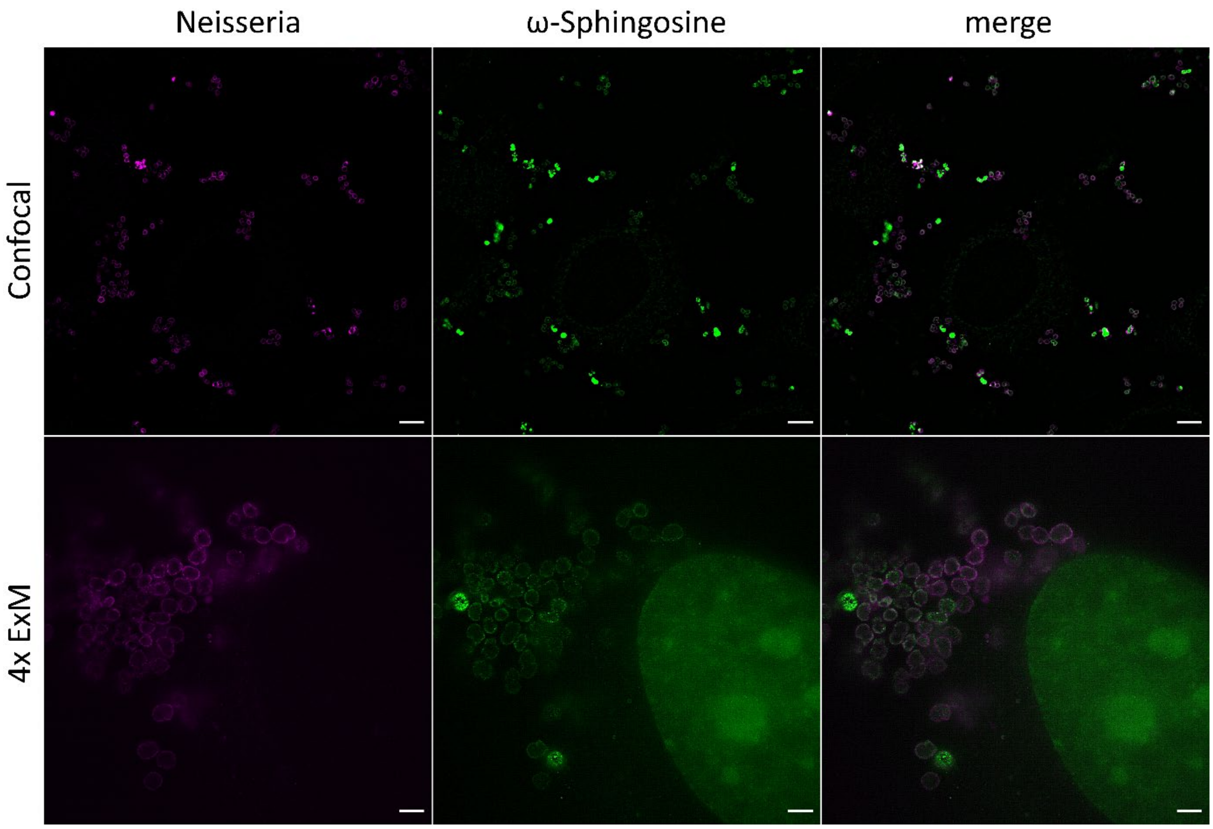

Supplementary Figure 22. Confocal (upper row) and 4x ExM-SIM images (lower row) of Chang cells infected with *Neisseria gonorrhoeae* for 4 h, fed with ω-sphingosine, fixed, permeabilized and stained with DIBO-Alexa Flour 488 (green) and anti-Neisseria (magenta). SIM images clearly show incorporation of ω-sphingosine into the membrane of Neisseria. The data were obtained from *n*=2 independent experiments. Scale bars, 5 µm (unexpanded confocal), 4 µm (expanded SIM).

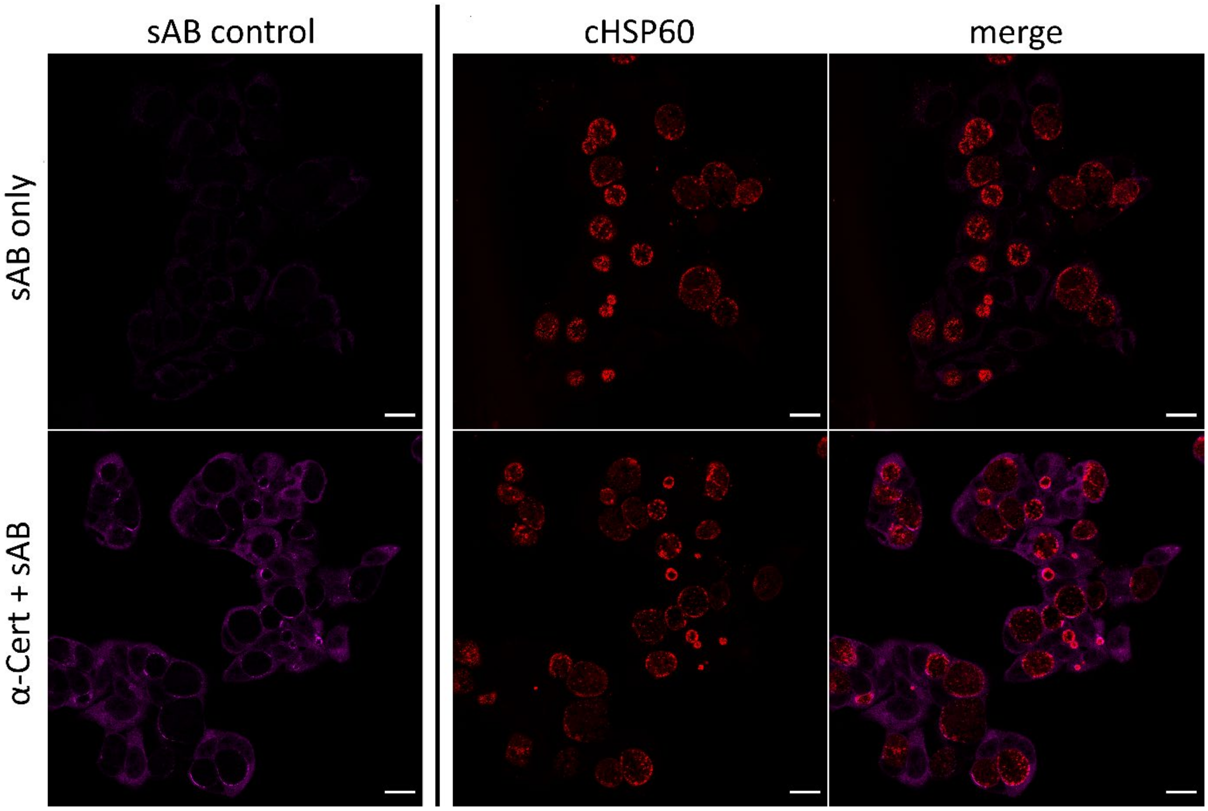

Supplementary Figure 23. Confocal images of unexpanded infected Hela229 cells infected with *Chlamydia trachomatis* for 24 h, fed with α-NH_2-ω-N_3-C_6-ceramide, fixed, permeabilized and immunolabeled for α-Cert with ATTO 647N (magenta) and chlamydial HSP60 with Cy3 (red). Labeling with secondary antibodies alone does not show any staining of chlamydial particles (sAB only/sAB control). The data were obtained from n=2 independent experiments. Scale bars, 20 µm.

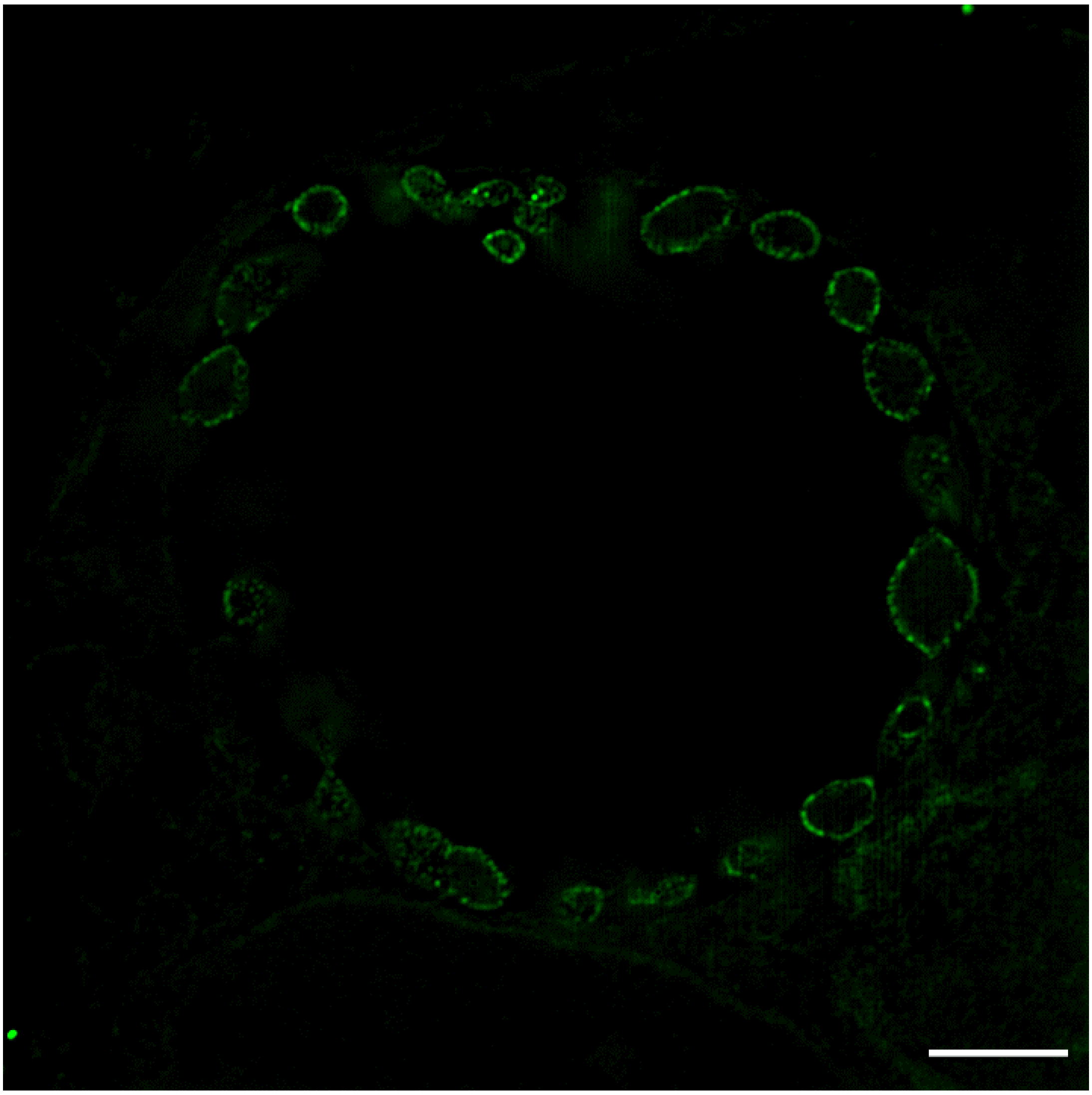

Supplementary Figure 24. 10x ExM-SIM image of Hela229 cells infected with *Chlamydia trachomatis* for 24 h, fed with α-NH_2-ω-N_3-C_6-ceramide, fixed, permeabilized and stained with DBCO-Alexa Fluor 488. Chlamydia are clearly located at the inclusion membrane. The data were obtained from *n*=5 independent experiments. Scale bar, 10 µm.

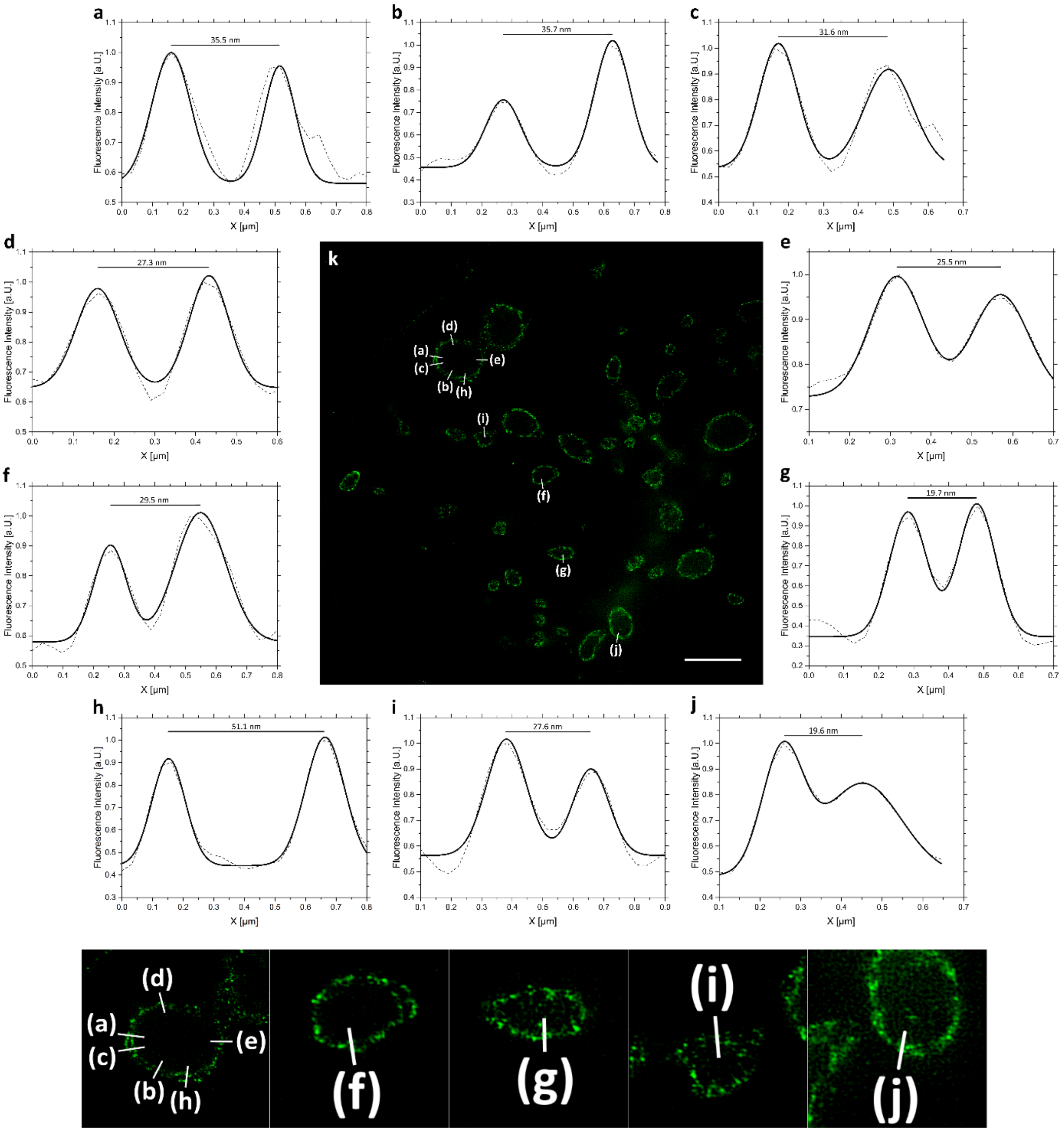

Supplementary Figure 25. Cross sectional intensity profiles (**a-j**) of 10x expanded *C. trachomatis* fed with α-NH_2-ω-N_3-C_6-Ceramide (green) in Hela229 cells. The distance between the inner and outer membrane varies between 19.7 nm and 51.1 nm. (k) SIM image of one of three 10x expanded sample used for data analysis.

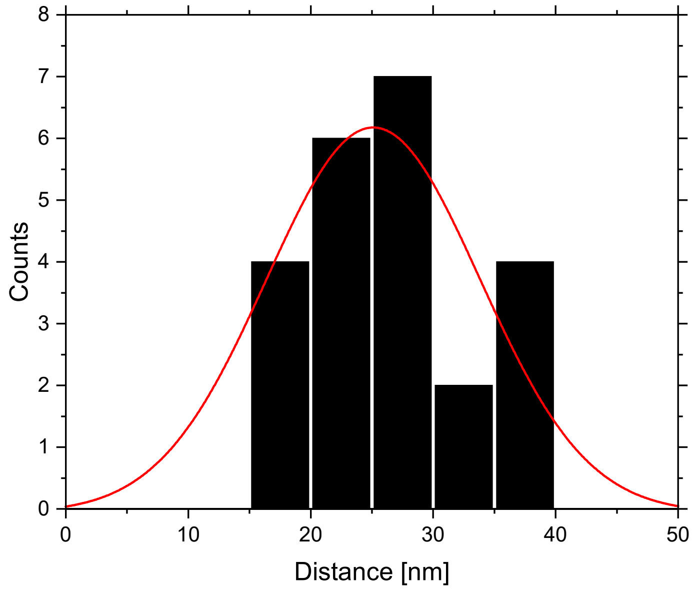

Supplementary Figure 26. Histogram and fit of 23 distances determined from cross sectional intensity profiles from three biological replicates infected with *C. trachomatis* and fed with α-NH_2-ω-N_3-C_6-Ceramide (Supplementary Figure S22). Only those bacteria were selected whose orientation allowed us to visualize spatially separated OM and IM (i.e. frontal views of bacteria) and determine the distance between the two membranes to 27.6 ± 7.7 nm (s.d.). Source data are provided as Source Data file.

References

[1] R.-B. Yan, F. Yang, Y. Wu, L.-H. Zhang, X.-S. Ye. An efficient and improved procedure for preparation of triflyl azide anmd application in catalytic diazotransfer reaction. *Tetrahedron Lett.* **46**, 8993–8995 (2005).

[2] Z. A. Wang, Y. Kurra, X. Wang, Y. Zeng, Y.-J. Lee, V. Sharma, H. Lin, S. Y. Dai, W. R. Liu. A Versatile Approach for Site-Specific Lysine Acylation in Proteins. *Angew. Chem. Int. Ed.* **56**, 1643–1647 (2017).

[3] O. I. Bol'shakov, I. O. Lebedyeva, A. R. Katritzky. 17α-ethynylestradiol peptide labeling by 'click' chemistry. *Synthesis* **44,** 2926–2932 (2012).

[4] L. Schoonen, J. Pille, A. Borrmann, R. J. M. Nolte, J. C. M. van Hest. Sortase A-Mediated N-Terminal Modification of Cowpea Chlorotic Mottle Virus for Highly Efficient Cargo Loading. *Bioconjug. Chem.* **26**, 2429–2434 (2015).

[5] C.-M. Chong, S. Gao, B.-Y. Chiang, W.-H. Hsu, T.-C. Lin, T.-C. Chen , C.-H. Lin. An acyloxymethyl ketone-based probe to monitor the activity of glutathionylspermidine amidase in Escherichia coli. *ChemBioChem* **12**, 2306–2309 (2011).
